# Atomically Precise Water‐Soluble Graphene Quantum Dot for Cancer Sonodynamic Therapy

**DOI:** 10.1002/advs.202105034

**Published:** 2022-01-17

**Authors:** Yang‐Yang Ju, Xiao‐Xiao Shi, Shu‐Yu Xu, Xiao‐Hui Ma, Rong‐Jing Wei, Hao Hou, Cheng‐Chao Chu, Di Sun, Gang Liu, Yuan‐Zhi Tan

**Affiliations:** ^1^ State Key Laboratory for Physical Chemistry of Solid Surfaces Department of Chemistry College of Chemistry and Chemical Engineering Xiamen University Xiamen 361005 China; ^2^ State Key Laboratory of Molecular Vaccinology and Molecular Diagnostics & Center for Molecular Imaging and Translational Medicine School of Public Health Xiamen University Xiamen 361005 China; ^3^ School of Chemistry and Chemical Engineering State Key Laboratory of Crystal Materials Shandong University Ji'nan 250100 China

**Keywords:** atomic precision, graphene quantum dot, sonodynamic therapy, water‐soluble

## Abstract

Although water‐soluble graphene quantum dots (GQDs) have shown various promising bio‐applications due to their intriguing optical and chemical properties, the large heterogeneity in compositions, sizes, and shapes of these GQDs hampers the better understanding of their structure‐properties correlation and further uses in terms of large‐scale manufacturing practices and safety concerns. It is shown here that a water‐soluble atomically‐precise GQD (WAGQD‐C_96_) is synthesized and exhibits a deep‐red emission and excellent sonodynamic sensitization. By decorating sterically hindered water‐soluble functional groups, WAGQD‐C_96_ can be monodispersed in water without further aggregation. The deep‐red emission of WAGQD‐C_96_ facilitates the tracking of its bio‐process, showing a good cell‐uptake and long‐time retention in tumor tissue. Compared to traditional molecular sonosensitizers, WAGQD‐C_96_ generates superior reactive oxygen species and demonstrates excellent tumor inhibition potency as an anti‐cancer sonosensitizer in in vivo studies. A good biosafety of WAGQD‐C_96_ is validated in both in vitro and *in* vivo assays.

## Graphical abstract

A water‐soluble atomically‐precise graphene quantum dot (WAGQD) with deep‐red emission is synthesized and shows a superior sensitizing potency for cancer sonodynamic therapy. The general synthetic strategy will enable the synthesis of WAGQDs with different sizes, edges, and tailored properties for further bio‐applications.

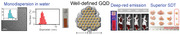

## 1 Introduction

Graphene quantum dots (GQDs), as a class of zero‐dimensional flat graphenic materials with lateral sizes smaller than 10 nm. Water‐soluble GQDs have been proven to be potential in various biological applications,^[^ ^1^ ^]^ such as bioimaging, sensing, and biological therapy. However, the large heterogeneity in compositions, sizes, and shapes of these water‐soluble GQDs results in significant challenges in better understanding their structure‐properties correlation and further clinical uses in terms of large‐scale manufacturing practices and safety concerns. On the other hand, GQDs with clear and precise structures can be synthesized by rational organic route using dehydrocyclization of dendritic polyphenylenes.^[^ ^2^ ^]^ These well‐defined organically‐synthesized GQDs have shown applications in electronics, optics, and energy conversion^[^ ^3^ ^]^ but are rarely used in biology,^[^ ^4^ ^]^ because most of them are naturally water‐insoluble and prone to aggregate into stacked large particles due to strong *π*—*π* interactions between the nano‐scale planar carbon skeleton. A few well‐defined warped GQDs functionalized by oligo‐ethylene‐glycol chains can be water‐soluble, due to the hindered *π*—*π* stacking between their warped skeleton,^[^ ^4^, ^5^ ^]^ however, they were still inclined to assemble into nano‐sized aggregations.^[^ ^5^ ^]^ A general synthetic strategy to make these atomically‐precise GQDs soluble and monodispersed in water is highly demanded, which can provide a series of well‐defined nanomaterials beyond the small molecules for bio‐applications, for example, sonodynamic therapy (SDT).

SDT is a non‐invasiveness and powerful strategy for anti‐cancer treatment.^[^ ^6^ ^]^ As a result, SDT has gained significant attention owing to its good penetration depth and fewer side effects of ultrasonic (US).^[^ ^7^ ^]^ SDT relies on sonomechanical and sonochemical processes induced by US irradiation to inhibit tumor growth.^[^ ^8^ ^]^ Compared to hydrodynamic stress and cavitation, the sonochemical process creates cytotoxic reactive oxygen species (ROS) to damage the tumor during SDT.^[^ ^9^ ^]^ More importantly, the sonochemical process could greatly be enhanced by adding US‐responsive sensitizers (sonosensitizers) to yield improved SDT potency.^[^ ^10^ ^]^ Therefore, a sonosensitizer with high ROS sensitizing is required during SDT. Most sonosensitizers are originally derived from photosensitizers^[^ ^10^, ^11^ ^]^ like porphyrin compounds, xanthone products, and antitumor drugs, among others. However, traditional molecular sonosensitizers exhibit relatively low ROS production efficiencies. Meanwhile, traditional molecular sonosensitizers suffer from ROS‐mediated self‐destruction, leading to chemical instability under US conditions.^[^ ^12^ ^]^ Nanomaterial‐based sonosensitizers,^[^ ^13^ ^]^ exhibit good stability and enhanced ROS generation. For example, the carbon nanomaterials could have a long excitation lifetime^[^ ^14^ ^]^ and multistate sensitization,^[^ ^15^ ^]^ that could enhance the ROS generation. However, despite the advantages of nanomaterial sonosensitizers in terms of potency, most lack well‐defined structures. The development of new sonosensitizers combining well‐defined structures of small molecules with superior sonosensitization efficacies of nanomaterials is crucial for the development of SDT.

Here we show a water‐soluble atomically precise GQD, namely WAGQD‐C_96_, was prepared by introducing sterically hindered water‐soluble functional groups. The well‐defined chemical and assembly structures of WAGQD‐C_96_ were characterized by mass, NMR, and UV–vis spectroscopies. The high‐resolution transmission electron microscopy (HRTEM) and dynamic light scattering (DLS) confirmed the monodispersion of WAGQD‐C_96_ in water without further aggregation. The biological behaviors of WAGQD‐C_96_, including cellular uptake, cellular SDT effects, and retention in tumor, were probed by its deep‐red emission. WAGQD‐C_96_ was proven to be able of generating more ROS under US irradiation than typical organic molecular sonosensitizers. In vivo studies indicated WAGQD‐C_96_ can greatly inhibit tumor growth in terms of SDT without obvious toxicity.

## 2 Results and Discussion

### 2.1 Synthesis and Characterization of WAGQD‐C_96_

**Figure 1:**
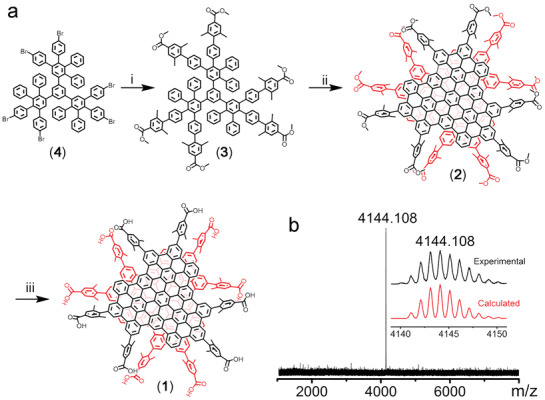
Synthesis of WAGQD‐C_96_. a) Synthetic route of 1. i) Pd_2_(bda)_3_, Sphos, Cs_2_CO_3_, toluene/H_2_O = 2/1, v/v, 100 °C, 16 h, yield: 67%. ii) DDQ, TfOH, DCM, 0 °C, 2 h, yield: 37%. iii) NaOH, THF/MeOH/H_2_O = 1/1/2, v/v/v, 60 °C, 24 h, yield: 92%. DCM: Dichloromethane, TfOH: Trifluoromethanesulfonic acid, Pd_2_(bda)_3_: tris(dibenzylideneacetone)dipalladium(0), THF: Tetrahydrofuran, MeOH: Methanol, Sphos: 2‐dicyclohexylphosphino‐2',6'‐dimethoxybiphenyl, DDQ: 2,3‐dichloro‐5,6‐dicyano‐1,4‐benzoquinone. b) mass spectra of 1. Inset shows that the isotopic distribution is in agreement with the calculated pattern.

The sterically hindered water‐soluble substituent, 4‐carboxy‐2,6‐dimethylphenyl, was introduced to the periphery of well‐defined planar GQDs to achieve WAGQD (**Figure** **1**). Accordingly, the ortho‐methyl groups of 4‐carboxy‐2,6‐dimethylphenyl can hinder the intermolecular *π*‐stacking of GQDs, while the para‐carboxyl would endow GQD with water‐solubility. Consequently, the strategy here to WAGQD can avoid the tedious synthesis of warped GQDs and in principle be able to be applied to different GQDs synthesized by organic bottom‐up methods. Following this blueprint, a dendritic phenylene precursor (3) containing 4‐(methoxycarbonyl)‐2,6‐dimethylphenyl was synthesized by the Suzuki coupling (Figure 1a, Figures S1–S7, Supporting Information). The resulting phenylene precursor was then transformed into functionalized C_96_ GQD (2) by dehydrocyclization using 2,3‐dichloro‐5,6‐dicyanobenzoquinone (DDQ) as an oxidant (Figure 1a). After separation by chromatography, compound 2 was obtained.

Similar to previously reported C_96_ GQD functionalized with mesityl groups,^[^ ^14^ ^]^ compound 2 also adopted a discrete bilayer structure without further intermolecular *π*—*π* stacking. This was validated by mass, NMR, and UV–vis spectroscopies (Figures S8–S10, Supporting Information). The formation of discrete bilayer structure is due to the balance between the stereo‐hindrance of peripheral substituents and strong *π*—*π* interactions of inner core.^[^ ^16^ ^]^ Next, the hydrolysis of the ester groups in substituents led to the formation of 4‐carboxy‐2,6‐dimethylphenyl functionalized C_96_ GQD (1) (Figure 1a). The mass spectrum of 1 showed a dominant peak at 4144.108 Da, confirming the preservation of the well‐defined bilayer structure after hydrolysis of ester groups (Figure 1b). Then, compound 1 was dissolved in water containing an equivalent of potassium carbonate, to produce the WAGQD‐C_96_ aqueous solution. The WAGQD‐C_96_ aqueous solution (pH = 7.3) was used for further photophysical and bio‐application studies.

**Figure 2:**
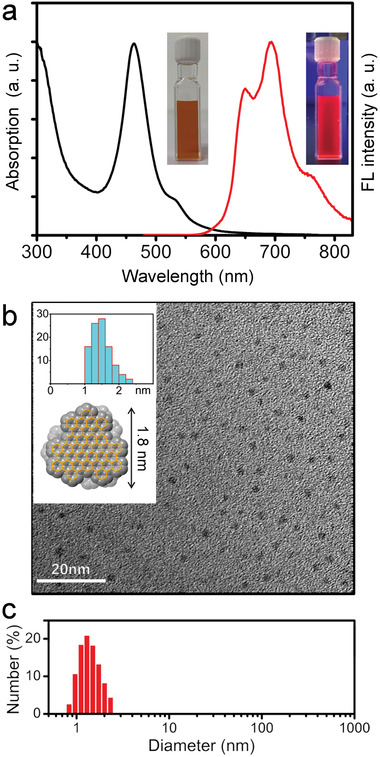
Characterization of WAGQD‐C_96_. a) UV–vis (black) and FL (red) spectra of WAGQD‐C_96_. Photographs of WAGQD‐C_96_ aqueous solution under ambient light (left) and a 365 nm UV lamp (right) are shown in the insert figures. b) HRTEM image of WAGQD‐C_96_. The inner graphenic core of WAGQD‐C_96_ and statistical size distribution based on HRTEM image are shown as the insert. c) DLS analysis of the WAGQD‐C_96_ aqueous solution.

As shown in **Figure** **2**a, the UV–vis spectrum of an aqueous solution of WAGQD‐C_96_ exhibited absorption bands at 463 and 528 nm, assigned to p and *α* absorption bands. WAGQD‐C_96_ revealed a deep red fluorescence (FL) with three peaks at 650, 694, and 760 nm, among which the second peak at 694 nm showed the highest intensity (Figure 2a). Such spectral features confirmed the H‐aggregated bilayer structure of WAGQD‐C_96_ without intermolecular *π*—*π* stacking under aqueous conditions. A typical Raman spectrum and IR spectrum (Figures S11 and S12, Supporting Information) confirmed the sp^2^ carbon skeleton of WAGQD‐C_96_. The morphology of WAGQD‐C_96_ in an aqueous solution was studied by HRTEM. As shown in Figure 2b, abundant uniform particles with a size of 1.6 ± 0.2 nm were observed (Figure 2b). Note that the particle size matched well with the diameter of graphenic core (1.8 nm) for a single WAGQD‐C_96_ (Figure 2b). Thus, particles observed by HRTEM imaging could be assigned to a single WAGQD‐C_96_ molecule, suggesting the monodispersed WAGQD‐C_96_ in water, in consistence with spectral characterizations. DLS analysis of WAGQD‐C_96_ aqueous solution showed a narrow size distribution (PDI = 0.21) with an average hydrodynamic radius (*R*_h_) of 1.5 nm (Figure 2c and Figure S13, Supporting Information), validating the monodispersion of WAGQD‐C_96_ in aqueous solution.

### 2.2 Fluorescent Imaging by WAGQD‐C_96_

**Figure 3:**
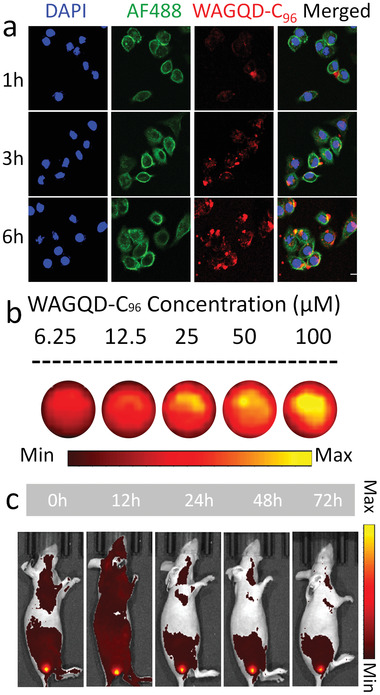
Fluorescent imaging by WAGQD‐C_96_. a) CLSM of HepG2 cells incubated with WAGQD‐C_96_ for 1, 3, and 6 h. Scale bar: 20 µm. b) FL images of WAGQD‐C_96_ with different concentrations. c) The in vivo FL images of HepG2‐tumor‐bearing model at different times after intratumor injection of WAGQD‐C_96_.

The water‐solubility of WAGQD‐C_96_ enables the studies on its biological behaviors, which can be conveniently probed by the deep‐red emission of WAGQD‐C_96_. In cellular level assays, confocal laser scanning microscopy (CLSM) was carried out and monitored, showing that WAGQD‐C_96_ presented time‐dependent cellular uptake (**Figure** **3**a). The flow cytometry analysis showed the similar cellular uptake of WAGQD‐C_96_ (Figure S14, Supporting Information). The deep‐red emission of WAGQD‐C_96_ can be also used for fluorescent diagnosis and visualization of tumors. As depicted in Figure 3b and Figure S15, Supporting Information, the FL images and calculated mean intensities indicated good photoemission and concentration‐dependent relationship of WAGQD‐C_96_. The time‐dependent retention of WAGQD‐C_96_ in the tumor region was then visually inspected after intratumoral injection. Long‐time retention of WAGQD‐C_96_ in tumor tissue was observed with signals mostly detained up to 72 h (Figure 3c and Figure S16, Supporting Information). All animal assays were carried out deferring to the protocols approved by Xiamen University Laboratory Animal Center (XMU2021101).

### 2.3 SDT Using WAGQD‐C_96_

**Figure 4:**
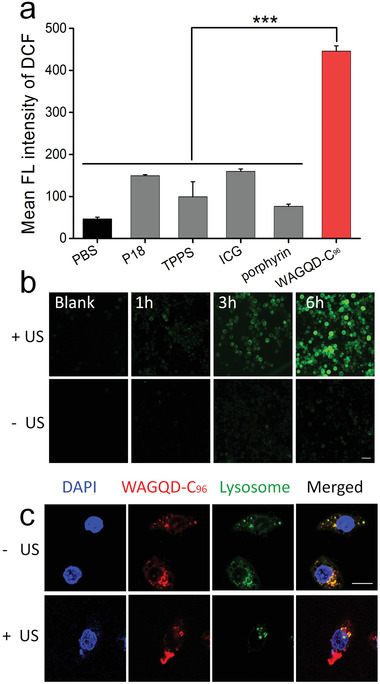
In vitro sonosensitization of WAGQD‐C_96_. a) ROS generated levels from WAGQD‐C_96_ and other sonosensitizer [purpurin 18 (P18), meso‐tetrakis (4‐sulfonatophenyl) porphyrin (TPPS), ICG, and porphyrin] at 20 µm concentration under ultrasound at 0.56 W cm^−2^ for 5 min. PBS: phosphate buffer saline. All data present as means ± SD (n = 3). P‐values are calculated using one‐way ANOVA with GraphPad Prism Software, ***p < 0.001. b) Cellular ROS generation of WAGQD‐C_96_ with/without ultrasound treatment at 0.56 W cm^−2^ for 5 min. Scale bar: 100 µm. c) Lysosome escape in the presence of WAGQD‐C_96_ with/without ultrasound treatment at 0.56 W cm^−2^ for 5 min. Scale bar: 20 µm.

Besides its deep‐red photo‐emission, the US‐responsive ROS sensitization capability of WAGQD‐C_96_ was evaluated using 2,7‐dichlorodihydrofluorescein diacetate (DCFH‐DA) as ROS probe. Various concentrations of WAGQD‐C_96_ (Figure S17, Supporting Information) generated increased ROS levels under ultrasound irradiation at 0.56 W cm^−2^ for 5 min. Compared to the reported well‐defined organic sonosensitizers like P18, meso‐tetrakis (4‐sulfonatophenyl) porphyrin (TPPS), indocyanine green (ICG), and porphyrin, WAGQD‐C_96_ exhibited superior performance toward the production of ROS (**Figure** **4**a). Meanwhile, sonosensitizers must ensure low dark cytotoxicity as well. A safe concentration of WAGQD‐C_96_ was determined by MTT assays as 20 µm (Figure S18, Supporting Information). Then the cellular level SDT effect of WAGQD‐C_96_ was evaluated. As shown in Figure 4b and Figure S19, Supporting Information, abundant cellular ROS after 6 h internalization was observed under ultrasound irradiation at 0.56 W cm^−2^ for 5 min. In lysosome escape results (Figure 4c), the US treatment was able of destroying lysosomes and promoting its escape due to ROS generation. This would be conducive to the toxic generation of ROS in cytoplasm, useful for directly attacking the nucleus.^[^ ^17^ ^]^ MTT assays were used to evaluate the SDT effect of WAGQD‐C_96_. Compared to WAGQD‐C_96_ without ultrasound treatment (Figure S20, Supporting Information), the cell viability of HepG2 cells significantly decreased with WAGQD‐C_96_ concentration after ultrasound treatment for 5 min at 0.56 W cm^−2^. A fairly low IC50 of 9.8 µm manifested a high SDT efficiency of WAGQD‐C_96_.

**Figure 5:**
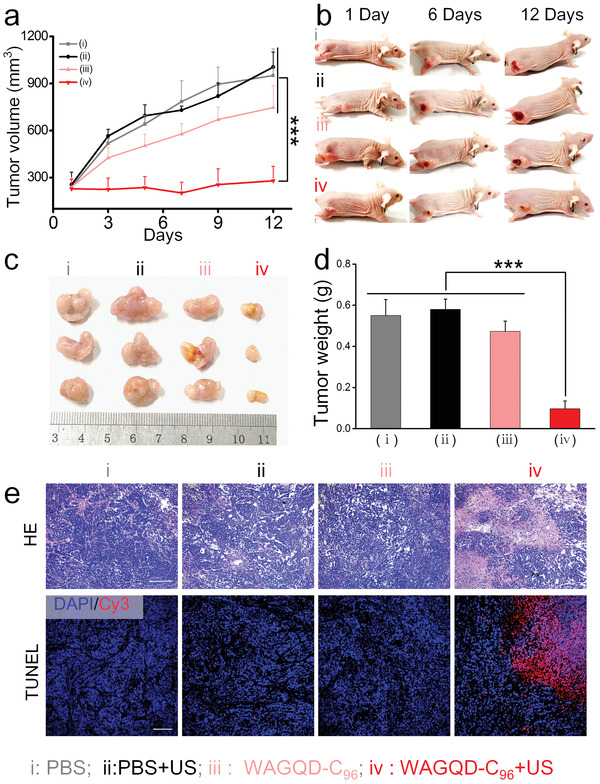
In vivo SDT using WAGQD‐C_96_. a) The tumor volume growth curves of different groups during 12‐days SDT and observation. All data present as means ± SD (n = 8). b) Tumor growth images during 12‐days SDT period. c) The ex vivo tumor images. d) The ex vivo tumor weight of different groups. All data present as means ± SD (n = 8). e) H&E and TUNEL staining images of different groups. Scale bar: 100 µm. P‐values are calculated using one‐way ANOVA with GraphPad Prism Software, ***p < 0.001.)

Encouraged by the excellent ROS performance and obvious SDT effect at the cellular level, the feasibility of WAGQD‐C_96_ as SDT reagent for antitumor was investigated in vivo. In view of the long‐term retention of WAGQD‐C_96_, we exploited two‐injection and four‐ultrasound irradiation for antitumor therapy. To this end, the volumes and images of tumors and body weights of four groups were recorded every two days during 12 consecutive days of therapy and observation period. As shown in **Figure** **5**a,b, no obvious tumor outburst was recorded after initial ultrasound treatment of WAGQD‐C_96_+ US group. By contrast, the tumor volumes in other control groups increased sharply, indicating that SDT induced by WAGQD‐C_96_ could dramatically eliminate the tumor. Also, WAGQD‐C_96_+ US groups displayed a superior tumor inhibitory rate of 70 % when compared to the other three groups (Figure S21, Supporting Information).

The anatomized tumor of WAGQD‐C_96_ under US irradiation also revealed the lowest values in both size and weight (Figure 5c,d) than other control groups. Next, pathological analyzes by H&E and TUNEL staining were performed to evaluate the ex vivo tumor tissues. As shown in Figure 5e, high tumor elimination and tumor cell apoptosis, including cell shrinkage and fragmentized nuclei were observed in WAGQD‐C_96_+ US group. Meanwhile, the body weights remained steady during the initial day, suggesting negligible physical toxicity of WAGQD‐C_96_ (Figure S22, Supporting Information). The ex vivo analysis of major organs, including the heart, liver, spleen, lung, and kidney, revealed no distinct damage, further confirming the biosafety of WAGQD‐C_96_ (Figure S23, Supporting Information). The hemanalysis also confirmed no obvious biotoxicity of WAGQD‐C_96_ during in vivo tests (Figure S24, Supporting Information).

## 3 Conclusion

In summary, a simple and general strategy was developed to obtain WAGQD by decorating sterically hindered water‐soluble functional groups on the well‐defined GQD core, which can address the long‐standing issue on the synthesis of monodispersed water‐soluble GQDs. WAGQD‐C_96_ here was characterized as a bilayer structure and can be monodispersed in water without further stacking, showing a well‐defined chemical and assembled structure. Probed by its deep‐red emission, good cellular uptake and long‐time retention in tumor were discovered. Both in vitro and in vivo experiments demonstrated the superior ROS sensitizing potency of WAGQD‐C_96_ under US irradiation coupled with good biosafety. Consequentially, WAGQD‐C_96_ can greatly inhibit tumor growth in in vivo SDT. Combined with the general synthetic strategy of WAGQD, other WAGQDs with different sizes, edges, and tailored properties can be developed for effective SDT, as well as for other bio‐applications.

## Conflict of Interest

The authors declare no conflict of interest.

## Supporting information

Supporting Information

## Data Availability

The data that support the findings of this study are available in the supplementary material of this article.
