# Supplementary material for: Atomically Precise Water‐Soluble Graphene Quantum Dot for Cancer Sonodynamic Therapy
Source: Adv Sci (Weinh). 2022 Jan 17;9(19):2105034. doi: 10.1002/advs.202105034 (PMC9259723; doi:10.1002/advs.202105034)
Supplement: Supplementary file 1 — Supporting Information [file ADVS-9-2105034-s001.pdf]

ADVANCED SCIENCE
Open Access

# Supporting Information

for *Adv. Sci.*, DOI 10.1002/advs.202105034

Atomically Precise Water-Soluble Graphene Quantum Dot for Cancer Sonodynamic Therapy

*Yang-Yang Ju, Xiao-Xiao Shi, Shu-Yu Xu, Xiao-Hui Ma, Rong-Jing Wei, Hao Hou, Cheng-Chao Chu, Di Sun, Gang Liu* and Yuan-Zhi Tan**

ADVANCED SCIENCE
Open Access

# Supporting Information

for *Adv. Sci.*, DOI: 10.1002/advs.202105034

## Atomically Precise Water-soluble Graphene Quantum Dot for Cancer Sonodynamic Therapy

*Yang-Yang Ju†, Xiao-Xiao Shi†, Shu-Yu Xu, Xiao-Hui Ma, Rong-Jing Wei, Hao Hou, Cheng-Chao Chu, Di Sun, Gang Liu*, Yuan-Zhi Tan**

Supporting Information

**Atomically Precise Water-soluble Graphene Quantum Dot for Cancer Sonodynamic Therapy**

*Yang-Yang Ju†, Xiao-Xiao Shi†, Shu-Yu Xu, Xiao-Hui Ma, Rong-Jing Wei, Hao Hou, Cheng-Chao Chu, Di Sun, Gang Liu*, Yuan-Zhi Tan**

† These authors contributed equally to this work.

Y. Ju, X. Ma, R. Wei, H. Hou, Prof. Y. Tan*
State Key Laboratory for Physical Chemistry of Solid Surfaces, Department of Chemistry, College of Chemistry and Chemical Engineering, Xiamen University, Xiamen, 361005, China
E-mail: yuanzhi_tan@xmu.edu.cn

X. Shi, S. Xu, C. Chu, Prof. G. Liu*
State Key Laboratory of Molecular Vaccinology and Molecular Diagnostics & Center for Molecular Imaging and Translational Medicine, School of Public Health, Xiamen University, Xiamen, 361005 China
E-mail: gangliu.cmitm@xmu.edu.cn

Prof. D. Sun
School of Chemistry and Chemical Engineering
State Key Laboratory of Crystal Materials
Shandong University
Ji'nan 250100, China.

# Contents

1. General information……………………………………………………………3

2. Synthetic details……………………………………………………………4-13

3. Biological experiments………………………………………………………14-22

4. Supporting reference…………………………………………………………….23

## 1. General information

Nuclear magnetic resonance (NMR) spectra were acquired on a Bruker AV 500/600 Spectrometer (Germany) at 298 K in the solvents indicated. Chemical shifts were represented in ppm units relative to TMS (0.00 ppm, $^1H$). Mass spectra (MS) were recorded on a Bruker time-of-flight mass spectrometer coupled with matrix-assisted laser desorption/ionization source (MALDI-TOF). Silica gel (300-400 mesh) was used for column chromatography. Absorption spectra were recorded using a Cary 5000 spectrometer. Fluorescence was measured on an Edinburgh FLS980 Fluorescence Spectrometer in the solvents indicated. Transmission electron microscopy (TEM, JEM-2100, JEOL, Japan) images of nanoparticles were obtained on air-dried ultra-thin carbon film. DLS measurements were performed using a Nano ZS 90 (Malvern, UK) instrument. All chemicals and solvents were purchased from commercial sources and used without further purification.

## 2. Synthetic details

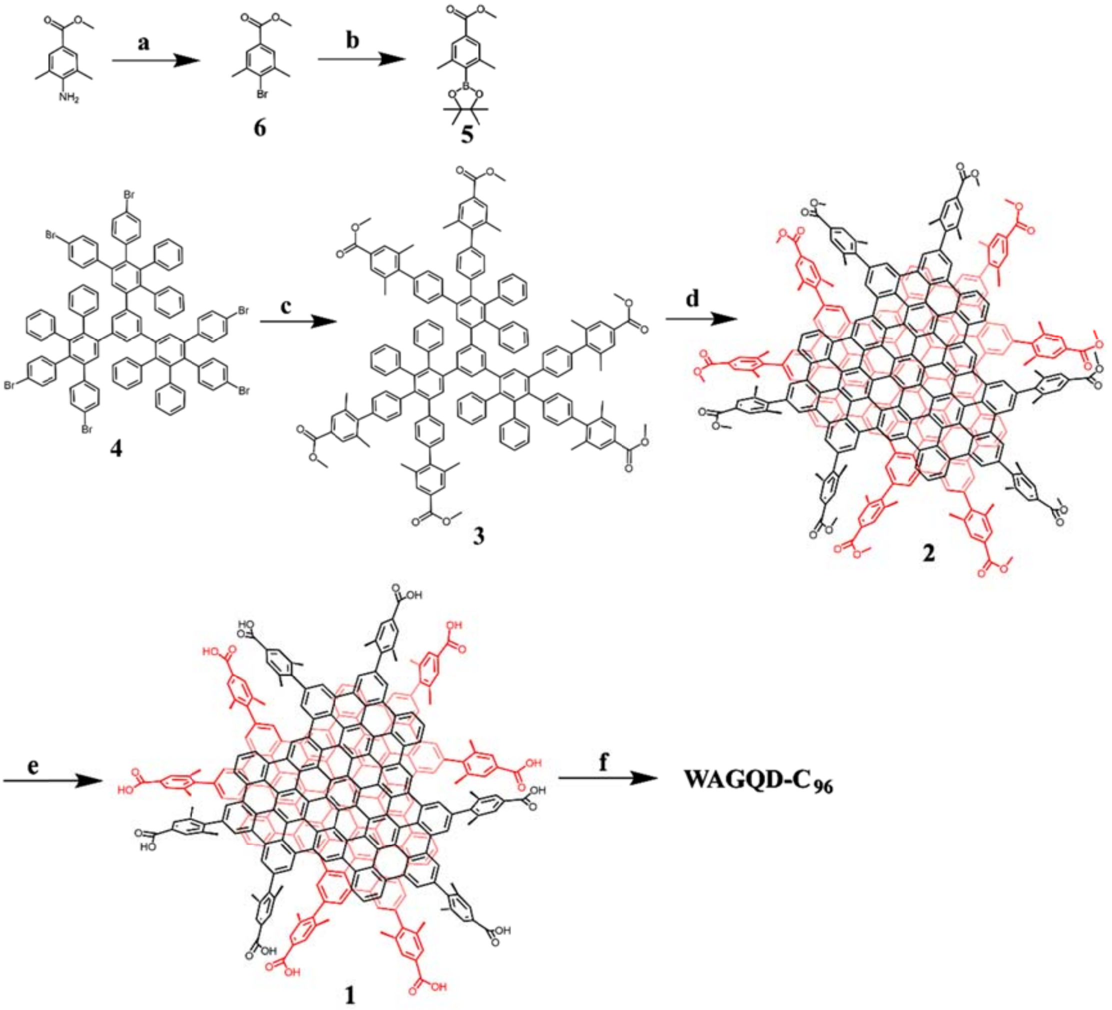


**Figure S1. Synthetic route of WAGQD-C$_{96}$.** Reagents and conditions: (a) TBAB, p-toluenesulfonic acid, Butyl nitrite, $CuBr_2$, Acetonitrile, 25 $^{o}$C, 0.5 h; (b) bis(pinacolato)diboron, $Pd_2$(bda)$_3$, Sphos, $Cs_2CO_3$, toluene/$H_2O$ = 2/1, v/v, 100 $^{o}$C, 20 h; (c) Compound 5, $Pd_2$(bda)$_3$, Sphos, $Cs_2CO_3$, toluene/$H_2O$ = 2/1, v/v, 100 $^{o}$C, 16 h; (d) DDQ, TfOH, DCM, 0 $^{o}$C, 2 h; (e) NaOH, THF/MeOH/$H_2O$ = 1/1/2, v/v/v, 60 $^{o}$C, 24 h; (f) $K_2CO_3$, $H_2O$, pH = 7-8. Abbreviation: THF: Tetrahydrofuran; $Pd_2$(bda)$_3$: tris(dibenzylideneacetone)dipalladium(0); MeOH: Methanol; Sphos: 2-dicyclohexylphosphino-2',6'-dimethoxybiphenyl; DCM: Dichloromethane; TBAB: tetrabutylammonium bromide; DDQ: 2,3-dichloro-5,6-dicyano-1,4-benzoquinone; TfOH: trifluoromethanesulfonic acid.

## Synthesis of Compound 6

A mixture of 4-amino-3,5-dimethylbenzoic acid methyl ester (5.40 g, 30 mmol), p-toluenesulfonic acid (6.20 g, 36.0 mmol), and tetrabutylammonium bromide (19.30 g, 60.0 mmol) was dissolved in acetonitrile (100 ml). After stirred for 10 min, butyl nitrite (3.70 g, 36 mmol) and $CuBr_2$ (67 mg, 0.30 mmol) were added. The reaction was stirred at room temperature for 0.5 h. Water (100 mL) was added and the resulting mixture was extracted with dichloromethane (50 mL×3). The combined organic layer was dried with $Mg_2SO_4$, filtered, and concentrated in vacuum. The crude product was then purified by silica gel column chromatography using petroleum ether/ethyl acetate (*v/v*, 5:1) as eluent to obtain **6** (6.30 g, 86 %) as a white solid. $^1$H NMR (500 MHz, $CDCl_3$) δ 7.71 (s, 2H), 3.90 (s, 3H), 2.44 (s, 6H) ppm. $^{13}$C NMR (125 MHz, $CDCl_3$) δ 166.78, 138.65, 133.03, 128.93, 128.36, 52.16, 23.86 ppm.

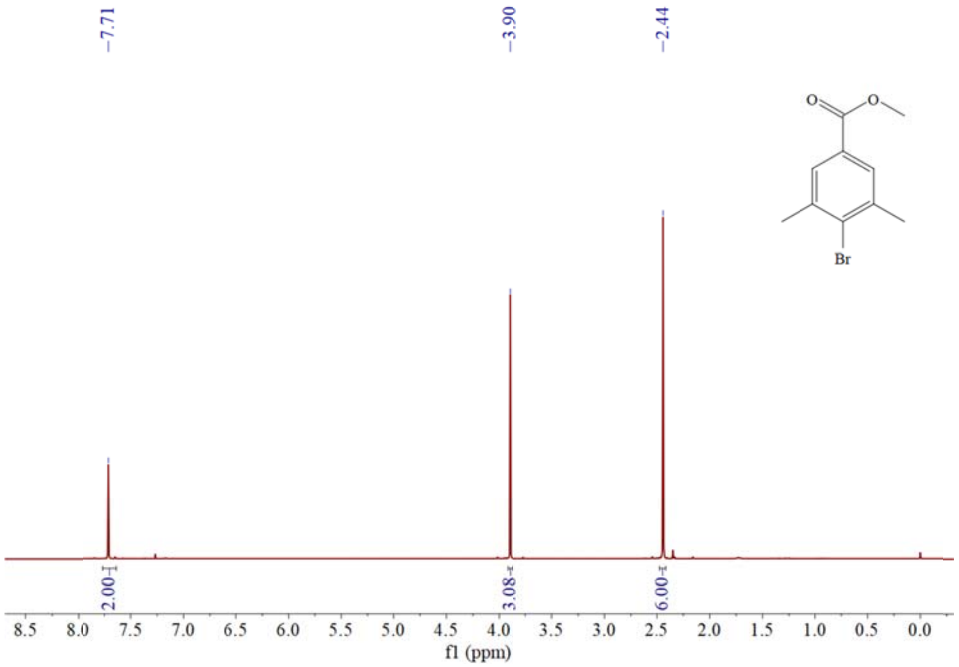


**Figure S2.** $^1$H NMR spectrum of Compound **6** (500 MHz, $CDCl_3$).

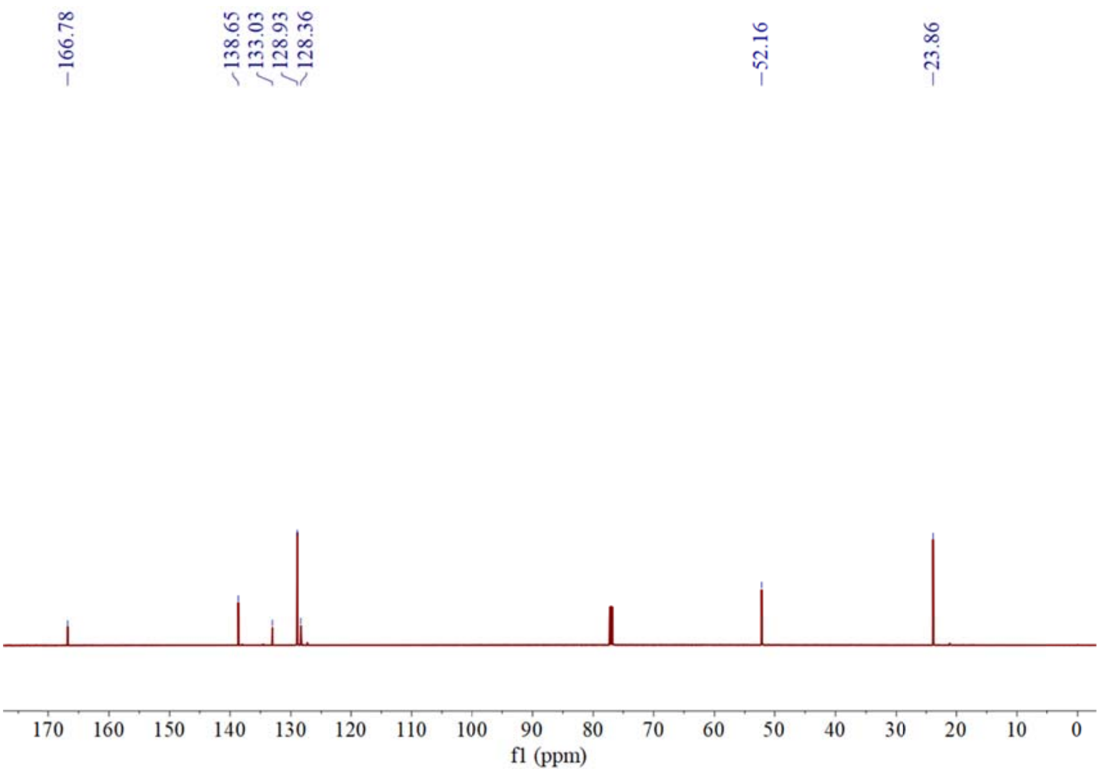


**Figure S3.** $^{13}C$ NMR spectrum of Compound **6** (125 MHz, $CDCl_3$).

**Synthesis of Compound 5**

Compound **6** (2.43 g, 10.0 mmol), bis(pinacolato)diboron (5.08 g, 20.0 mmol), $Pd_2(bda)_3$ (452 mg, 0.50 mmol), Sphos (405 mg, 0.10 mmol) and $Cs_2CO_3$ (8.15 g, 25.0 mmol) were mixed in toluene (50 mL) and water (25 mL), which was stirred at 100 °C under $N_2$ for 20 h. After cooled to RT, the reaction mixture was filtered and washed with ethyl acetate. After removing the solvent under reduced pressure, the residue was purified by silica gel column chromatography (petroleum ether/ethyl acetate = 5/1, *v/v*), offering **5** (2.32 g, 80 %) as a white solid. $^1H$ NMR (500 MHz, $CDCl_3$) δ 7.62 (s, 2H), 3.87 (s, 3H), 2.43 (s, 6H), 1.38 (s, 12H) ppm. $^{13}C$ NMR (125 MHz, $CDCl_3$) δ 167.38, 141.90, 130.46, 127.18, 84.05, 51.87, 24.91, 22.03 ppm.

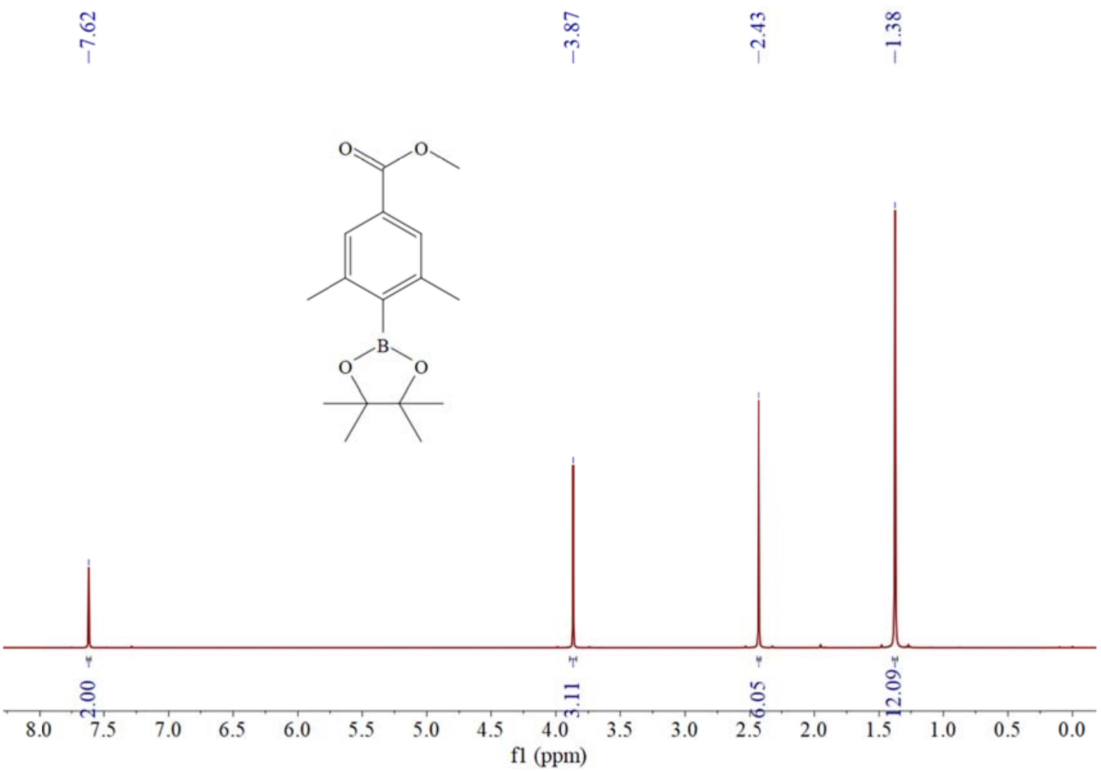


**Figure S4.** $^{1}H$ NMR of Compound **5** (500 MHz, $CDCl_3$).

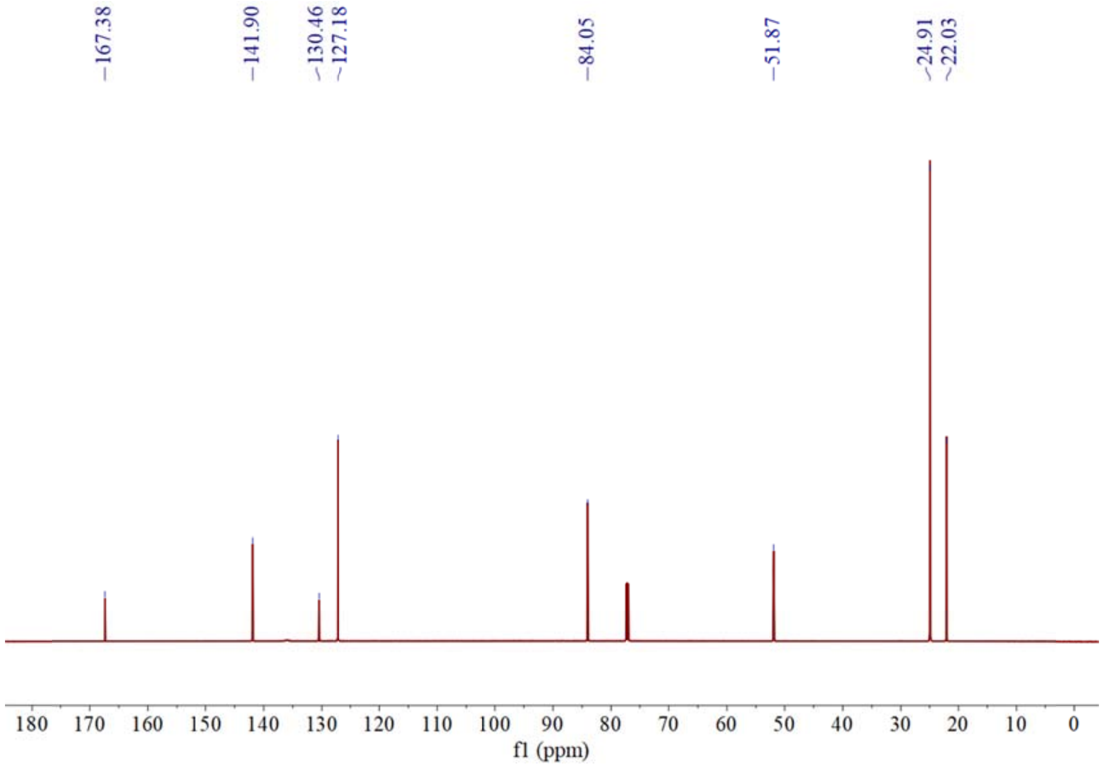


**Figure S5.** $^{13}C$ NMR of Compound **5** (125 MHz, $CDCl_3$)

## Synthesis of Compound 3

Compound **4** was prepared according to the reported literature.[1]

A mixture of Compound **4** (490 mg, 0.29 mmol), Compound **5** (1.50 g, 5.21 mmol), $Pd_2(bda)_3$ (159 mg, 0.174 mmol), Sphos (143 mg, 0.348 mmol) and $Cs_2CO_3$ (1.70 g, 5.21 mmol) in toluene (10 mL) in water (5.0 mL) was stirred at 100 $^oC$ for 16 h under $N_2$. Then the mixture was filtered and washed with ethyl acetate. The crude products were concentrated under reduced pressure and purified by silica gel column chromatography using petroleum ether/ ethyl acetate (*v/v*, 3:1) as the eluent, affording Compound **3** (425 mg, 67 %) as a white solid. $^1H$ NMR (600 MHz, $CD_2Cl_2/CS_2$) δ 7.70-7.66 (m, 12H), 7.23-7.20 (m, 9H), 7.18-7.16 (m, 6H), 6.99-6.97 (m, 9H), 6.93-6.85 (m, 18H), 6.76 (d, *J*=10.2 Hz, 6H), 6.71 (d, *J*=9.6 Hz, 6H), 6.63 (d, *J*=9.6 Hz, 6H), 3.86 (d, *J*=3.0 Hz, 18H), 1.92 (s, 9H), 1.84 (d, *J*=6.6 Hz, 18H), 1.81 (s, 9H) ppm. $^{13}C$ NMR (150 MHz, $CD_2Cl_2/CS_2$) δ 166.88, 146.45, 146.43, 141.40, 141.38, 140.85, 140.65, 140.54, 139.99, 139.36, 139.27, 139.07, 138.98, 137.59, 137.28, 136.56, 136.49, 136.15, 136.11, 131.99, 131.91, 131.87, 131.46, 130.25, 129.94, 128,64, 128.59, 128.22, 128.18, 128.14, 127.48, 127.13, 127.11, 126.79, 126.43, 125.86, 51.76, 51.75, 20.43, 20.39 ppm; HRMS (MALDI-TOF) m/z calcd for $C_{156}H_{126}O_{12}$ $[M]^+$: 2191.928, found: 2191.927.

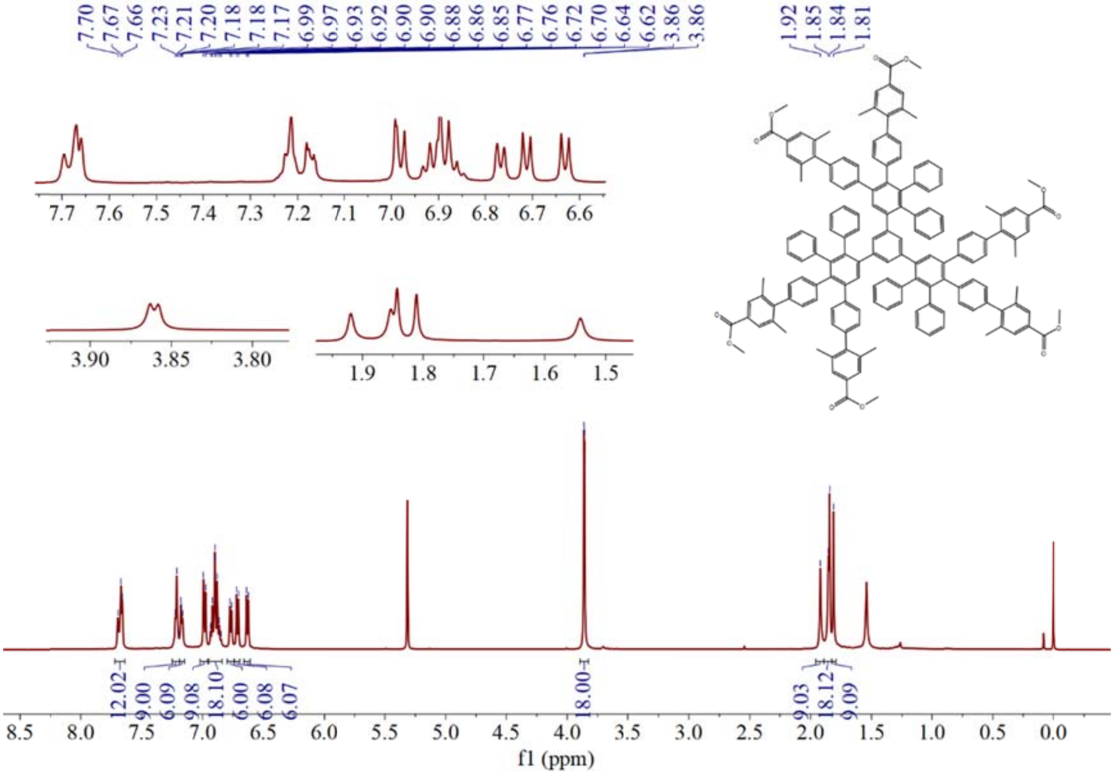


**Figure S6.** $^1H$ NMR of Compound **3** (600 MHz, $CD_2Cl_2/CS_2$).

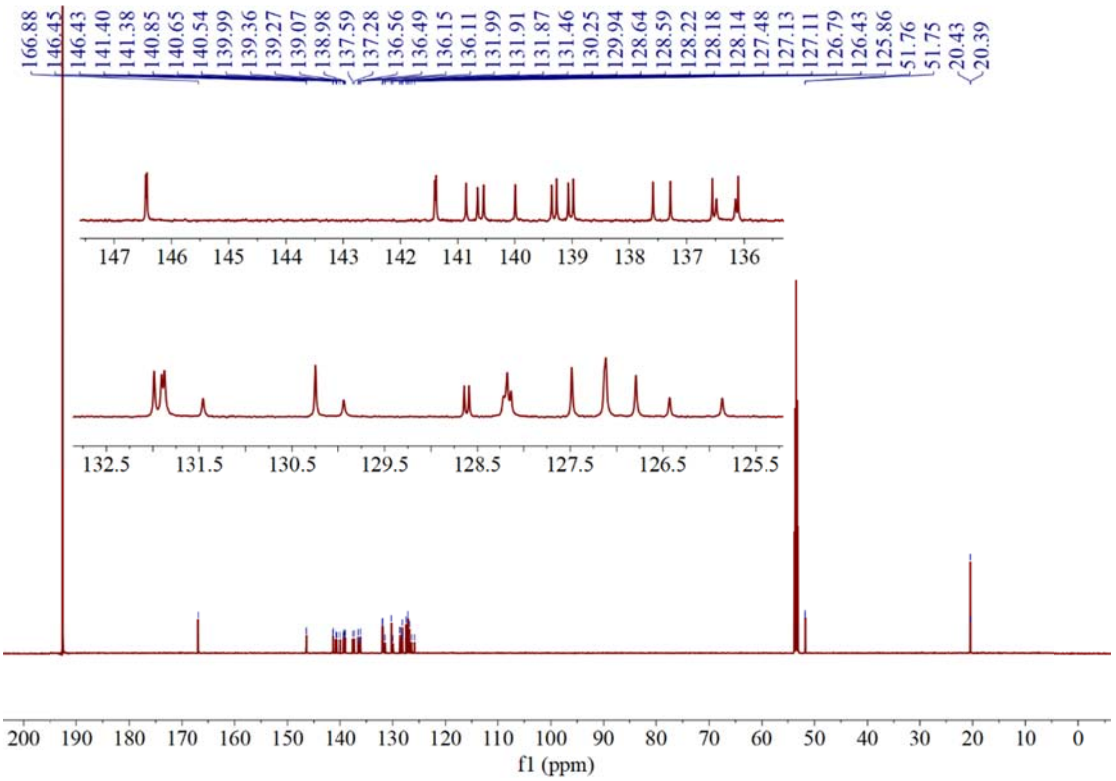


**Figure S7.** $^{13}C$ NMR of Compound **3** (150 MHz, $CD_2Cl_2/CS_2$).

## Synthesis of Compound 2

The anhydrous DCM solution (50 mL) of **3** (50 mg, 0.023 mmol) and DDQ (186 mg, 0.82 mmol) was added 0.2 mL of TfOH under $N_2$ at 0 °C. The reaction mixture was stirred at 0 °C for 2 h and quenched by triethylamine. Then the reaction mixture was washed with water (50 mL × 3). The organic phase was dried over anhydrous $Na_2SO_4$ and concentrated under reduced pressure. The residue was purified by silica gel column chromatography using dichloromethane/tetrahydrofuran (*v/v*, 20:1) as the eluent, affording Compound **2** (18 mg, 37 %) as a red solid. $^1H$ NMR (600 MHz, $CDCl_3$) δ 11.17 (s, 6H), 10.28 (s, 6H), 9.64 (s, 6H), 9.31 (s, 6H), 9.28 (d, $J$=10.2 Hz, 6H), 9.10 (d, $J$=7.2 Hz, 12H), 8.37 (s, 6H), 8.29 (s, 12H), 8.26-8.23 (m, 6H), 8.02 (s, 6H), 4.36 (s, 18H), 4.14 (s, 18H), 3.44 (s, 18H), 3.37 (s, 18H), 2.38 (s, 18H), 2.03 (s, 18H) ppm; HRMS (MALDI-TOF) m/z calcd for $(C_{156}H_{90}O_{12})_2$ $[M]^+$: 4312.296, found: 4312.295.

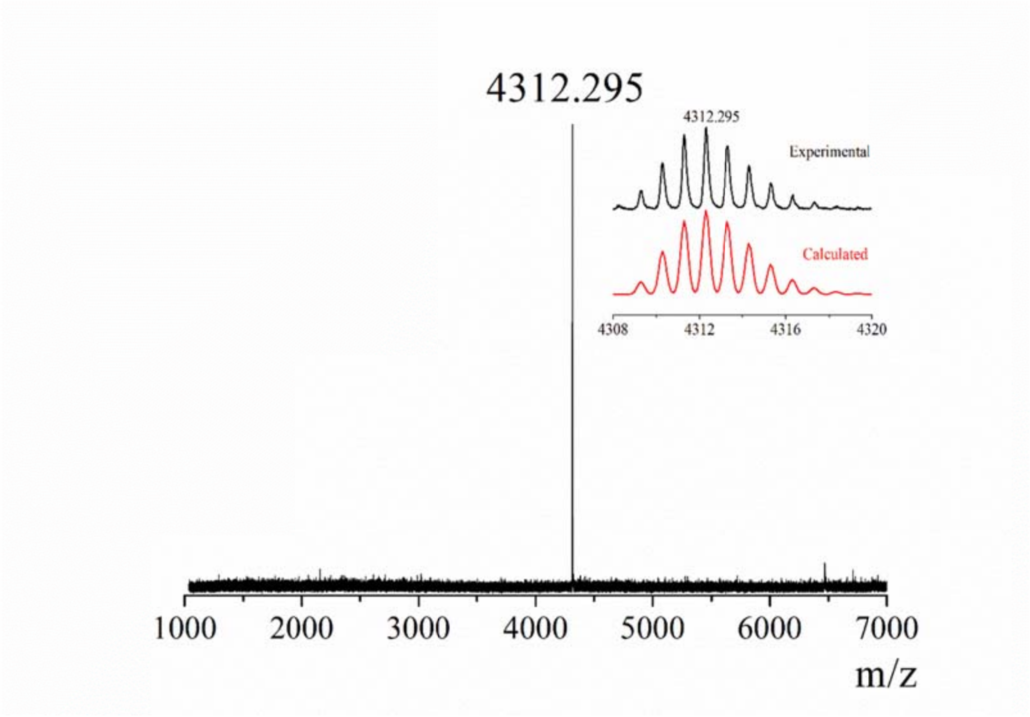


**Figure S8.** MALDI-TOF MS spectrum of Compound **2**. Inset shows that the isotopic distribution is in agreement with the simulated pattern. The single peak observed at 4312.295 Da matches the formula of dimeric $[C_{96}H_{24}(C_6H_2CH_3CH_3COOCH_3)_6]_2$, which reveals the bilayer stacked structure of Compound **2**.

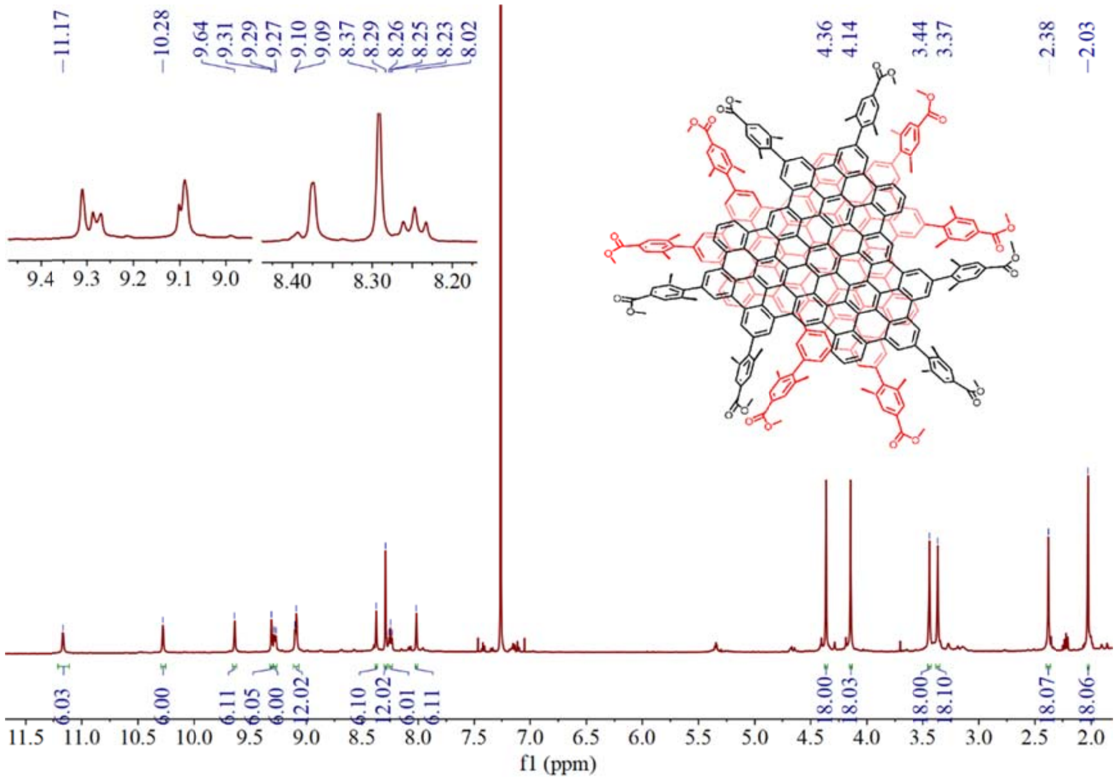


**Figure S9.** $^{1}$H NMR of Compound **2** (600 MHz, $CDCl_3$). Four $^{1}$H signals of

ortho-methyl groups and two $^{1}H$ signals of methoxy groups in 4-methoxycarbonyl-2,6-dimethylphenyl were observed, due to the breaking of mirror symmetry in the bilayer stacked structure,[2] which confirmed the bilayer structure of Compound **2** in solution.

### Synthesis of Compound 1

To a suspension of **2** (100 mg, 0.023 mmol) in 20 mL THF/MeOH/$H_2O$ (5/5/10 mL), NaOH (55 mg, 1.38 mmol) was added. The mixture was stirred at 60 °C for 24 h. After the pH value was adjusted to about 2-3 using 2 M HCl, the crude products were precipitated and collected by filtration. Then the precipitation was washed with water and dried under vacuum to afford **1** as a red solid (90 mg, 92 %). HRMS (MALDI-TOF) m/z calcd for $(C_{150}H_{78}O_{12})_2$ $[M]^+$: 4144.108, found: 4144.108.

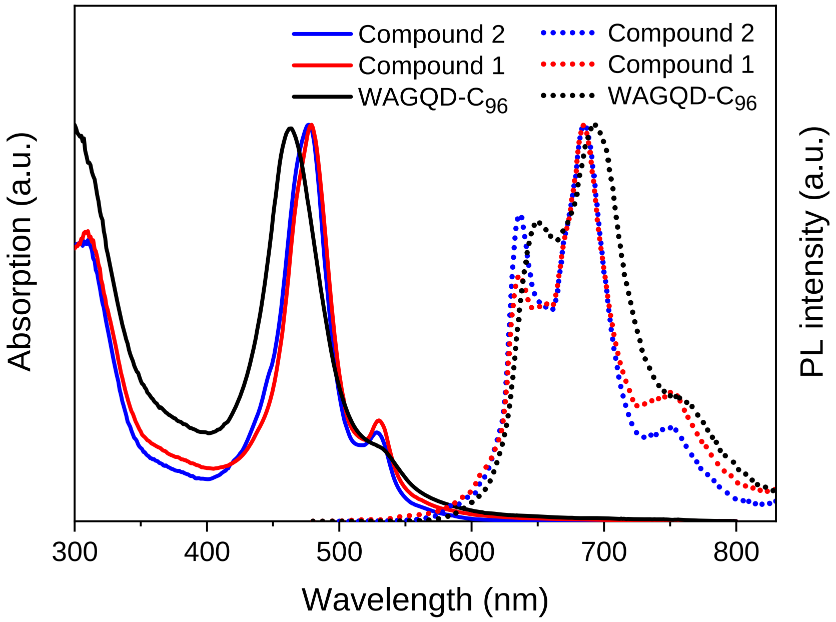


**Figure S10.** Absorption (solid lines) and emission spectra (dashed lines) of Compound **2** (in THF), Compound **1** (in THF), and WAGQD-$C_{96}$ (in water). The quantum yield for Compound 2 (in THF), Compound 1 (in THF) and WAGQD-$C_{96}$ (in water) was measured to be 7%, 8% and 1%, respectively, by absolute method using an integrating sphere.

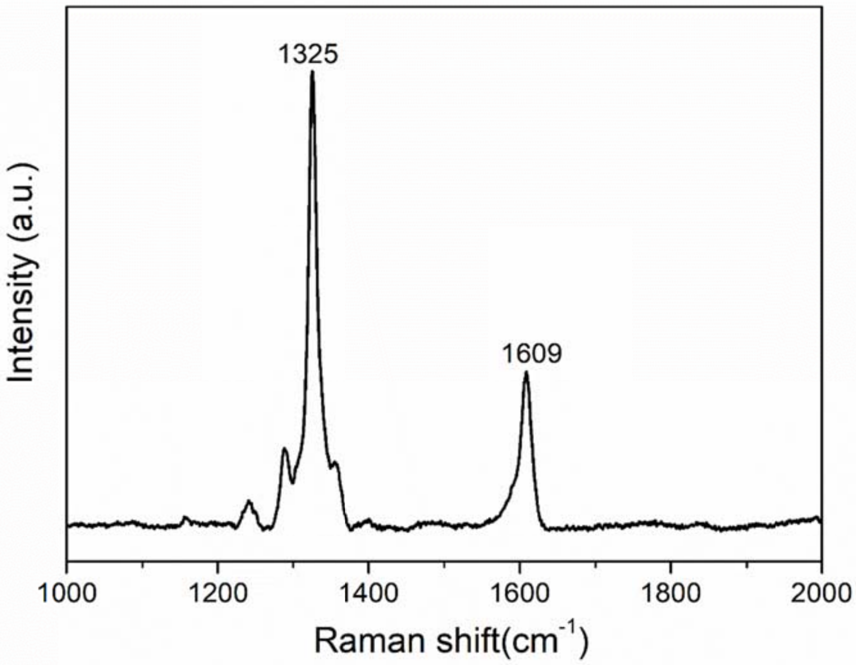


Figure S11. Raman spectrum of WAGQD-$C_{96}$ acquired by drop-costing WAGQD-$C_{96}$ aqueous solution on the silicon wafer.

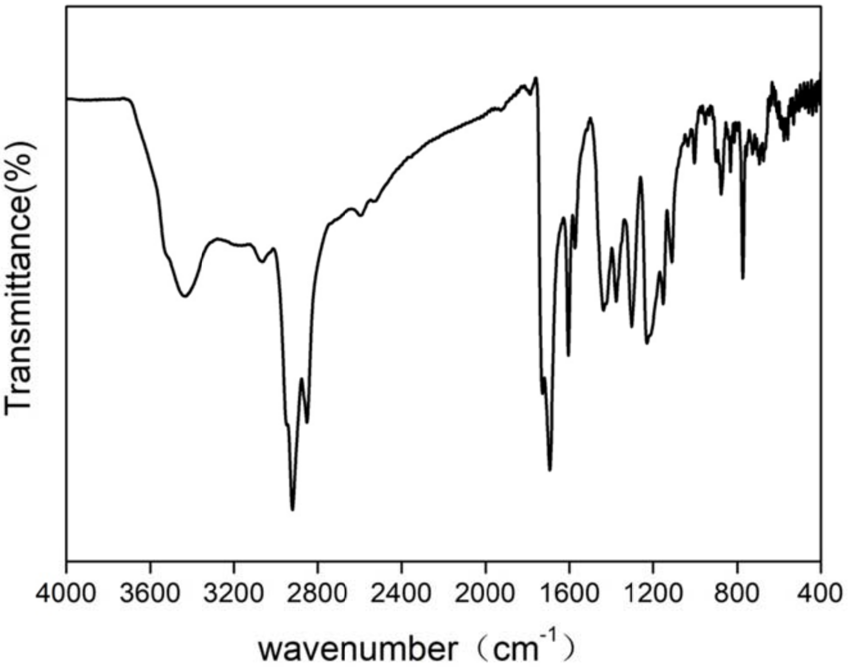


Figure S12. IR spectrum of WAGQD-$C_{96}$ using the sample prepared by freeze-drying of WAGQD-$C_{96}$ aqueous solution.

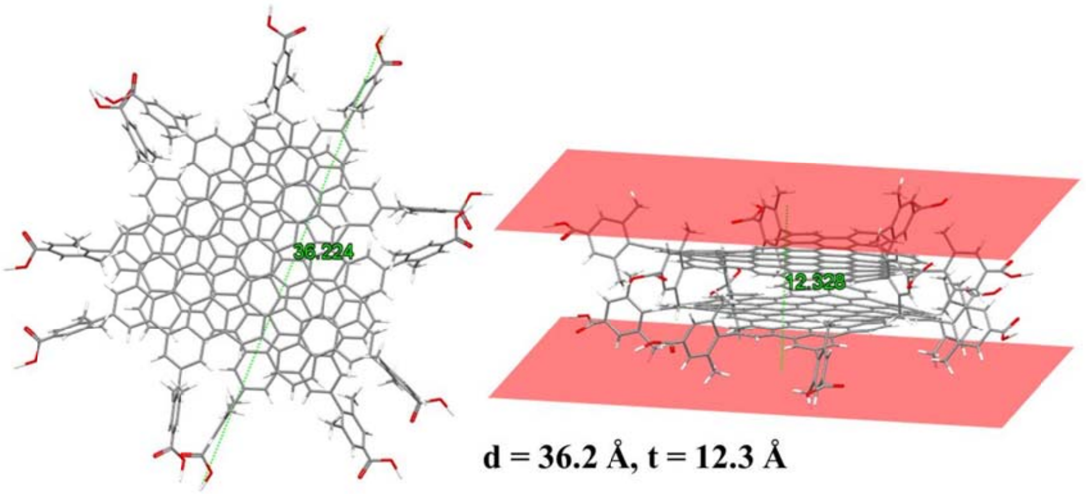


$$R_h = \frac{3}{4} d \left[ \sqrt{1 + \left(\frac{t}{d}\right)^2} + \frac{d}{t} ln\left(\frac{t}{d} + \sqrt{1 + \left(\frac{t}{d}\right)^2}\right) - \frac{t}{d} \right]^{-1} \qquad \textbf{\textit{(1)}}$$

Figure S13. Expected hydrodynamic radius ($R_h$) of WAGQD-$C_{96}$. WAGQD-$C_{96}$ has a planar and nonspherical structure, thus its expected hydrodynamic radius ($R_h$) can be converted from its lateral size (d) and thickness (t) through Equation (1).[3] The $R_h$ was calculated to be 16 Å for WAGQD-$C_{96}$, close to our measurement in DLS.

## 3. Biological experiments

**Detection of ROS generation**. The ROS generation from WAGQD-$C_{96}$ was detected by 2,7-Dichlorodihydrofluorescein diacetate (DCFH-DA) probe. Indeed, equivoluminal WAGQD-$C_{96}$ with different concentrations and DCFH-DA solutions were dealt with ultrasonic wave (0.56 W/cm$^2$, 5 min). The fluorescence intensity of DCF oxidized by reactive oxygen oxide was measured by Microplate Reader (Ex: 488 nm, Em: 525 nm). Likewise, ROS production levels from WAGQD-$C_{96}$ and various commercialized sonosensitizers (Purpurin 18, TPPS, ICG and porphyrin) were evaluated. The intercellular ROS levels were similarly detected using DCFH-DA probe by CLSM (Olympus FV1200) against WAGQD-$C_{96}$.

**Cellular uptake.** Firstly, the dark toxicity of WAGQD-$C_{96}$ with different concentrations against HepG2 cells was assessed by MTT assay, which the result indicated WAGQD-$C_{96}$ at 20 μM concentration was safe that the cell viability of HepG2 cells was more than 90 %. HepG2 cells were seeded and incubated in 6-well plates (1×10$^5$ /well) at 37 $^o$C for 12 h. And WAGQD-$C_{96}$ at the concentration of 20 μM medium was added to the cells for 1, 3 and 6 h incubation. At the predetermined point time, the cells were suspended in 0.5 mL PBS after digestion and centrifugation. The suspended cells were tested by flow cytometry (Beckman Coulter CytoFLEX) against APC-H channel. Furthermore, at 1, 3 and 6 h incubation, the fluorescent images of WAGQD-$C_{96}$ were recorded by CLSM (Zeiss 880) to verify the cellular uptake effect. The results are expressed as mean ± SD (n = 3).

**MTT assay.** The HepG2 tumor cells were incubated in 96-well plates (1×10$^4$ cells/well) for 12 h. The WAGQD-$C_{96}$ medium at the concentration of 0, 1, 5 and 10 μg/mL was replaced to cells for 6 h incubation, respectively. Then the cells were dealt with/without ultrasound treatment (0.56 W/cm$^2$, 5 min) and incubated for another 18 h. Next, the cells after washing were added with MTT agents (20 μL) and incubated for another 4 h, and DMSO (180 μL) was added and shook for 10 min. The cells were detected by Microplate Reader at 490 nm. The results are expressed as mean ± SD (n = 4).

**Animal model.** All animal assays were carried out deferring to the protocols approved by Xiamen University Laboratory Animal Center. The female nude mice (5-6 weeks, 20 g) were purchased from Academy of Military Medical Sciences (Beijing, China). The female subcutaneous tumor model of nude mice was constructed through r-thigh was injected with high-density HepG2 cells. These nude mice bearing subcutaneous tumor were used for subsequent *in vivo* experiments for one week after modeling.

**In vivo fluorescence imaging.** To investigate the fluorescence imaging capacity and retention time of WAGQD-$C_{96}$, the nude mice bearing subcutaneous tumor were injected with WAGQD-$C_{96}$ at the dose of 50 μL (175 μM) via intratumor injection, respectively. At the special time points, the fluorescence signals of tumor nude mice were observed in tumor location by IVIS Lumina imaging system. Furthermore, after 24 h of drug injection, the ex vivo fluorescence signal of the tumor and major organs was further observed to determine the drug distribution.

**In vivo SDT effects.** All groups (n=8) including PBS, PBS+US, WAGQD-$C_{96}$ and WAGQD-$C_{96}$+US (175 μM, 50 μL) were injected into female nude mice bearing subcutaneous HepG2 tumor via intratumor injection for every four days and total two times. After drug administration, the tumors were treated with ultrasonic wave (0.56 W/cm$^2$, 5 min) at 0 h and 48 h. And the tumor growth and body weight of nude mice were recorded every two days. After the therapy ends, the tumor and major organs of all groups were exsomatized for the histological analysis of H&E and TUNEL staining to assess the antitumor efficacy of sonodynamic therapy.

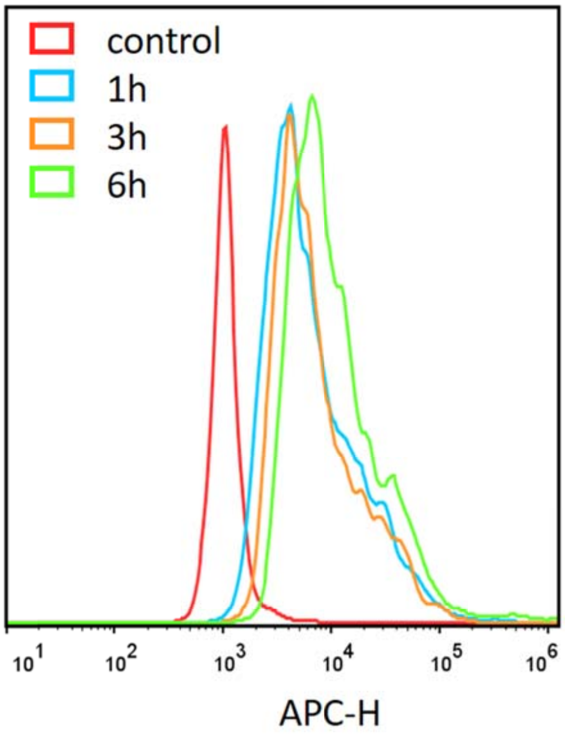


**Figure S14.** Flow cytometry of HepG2 cells incubated with WAGQD-$C_{96}$ for 1, 3 and 6 h. All data present as means ± SD (n = 3).

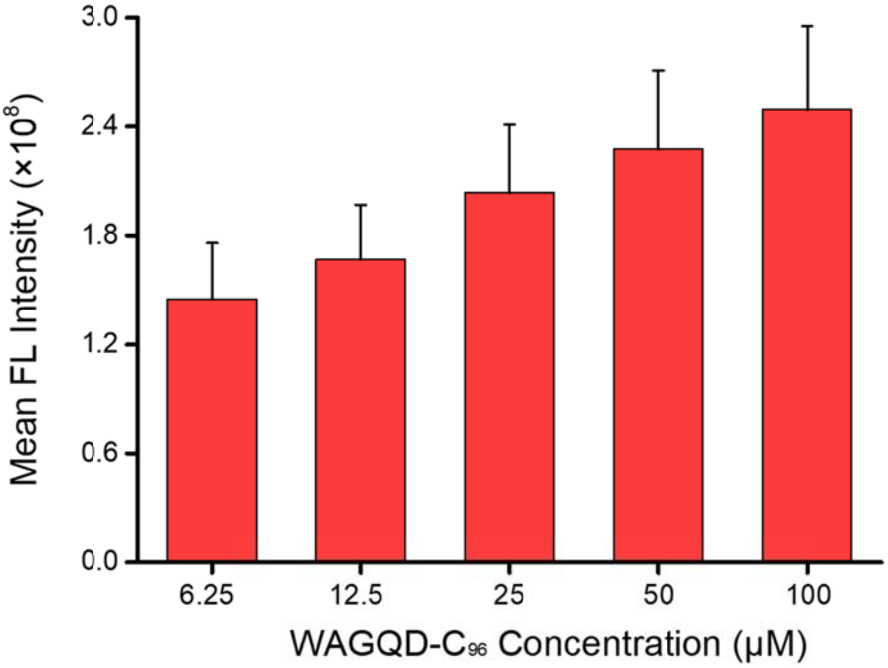


**Figure S15.** The mean FL intensity of WAGQD-$C_{96}$ with various concentrations. All data present as means ± SD (n = 3).

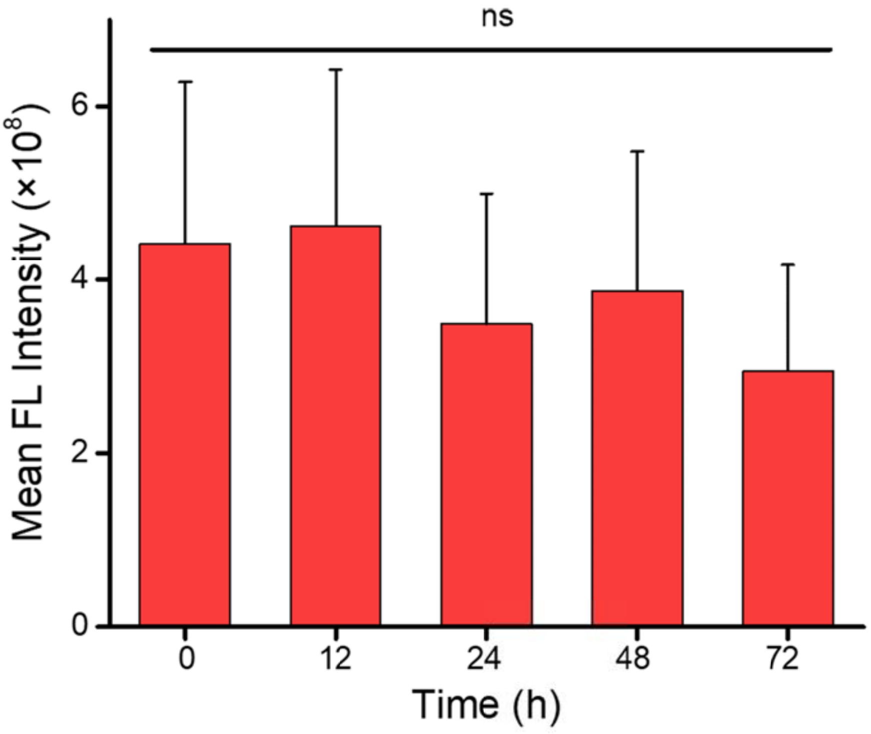


**Figure S16.** Mean FL intensity of tumor in vivo at different time. P-values are calculated using one-way ANOVA with GraphPad Prism Software, ns: *p* >0.05.

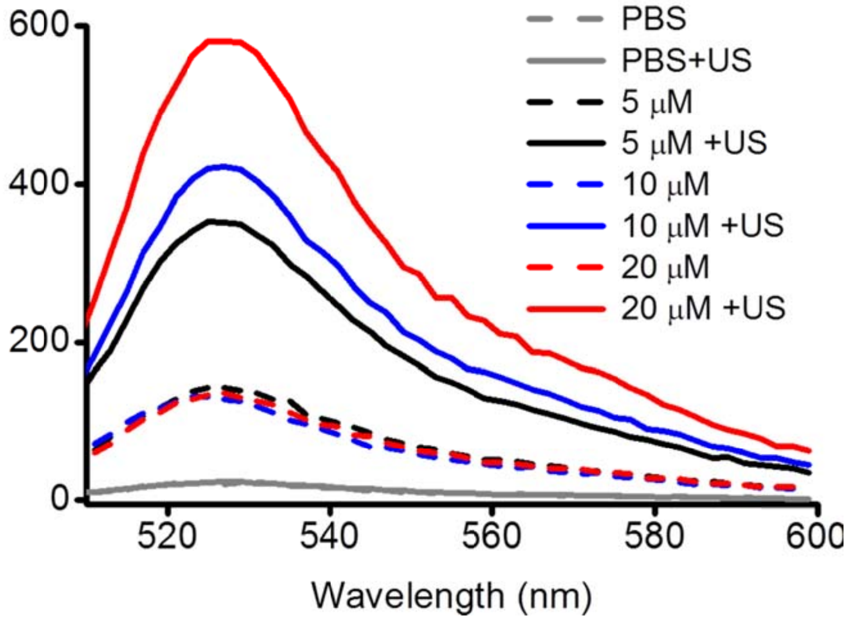


**Figure S17.** ROS generation of WAGQD-$C_{96}$ with different concentrations probed by DCFH-DA with/without ultrasound.

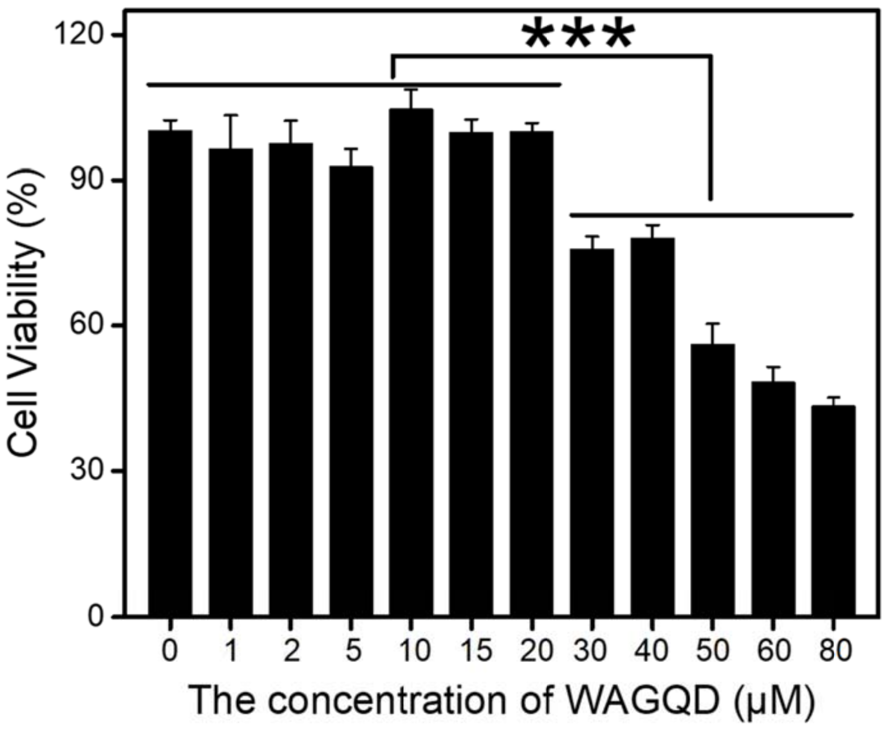


**Figure S18.** Cell viability of HepG2 cells incubated with different concentrations of WAGQD-$C_{96}$ for 24 h. All data present as means ± SD (n = 4). P-values are calculated using one-way ANOVA with GraphPad Prism Software, ***, $p < 0.001$.

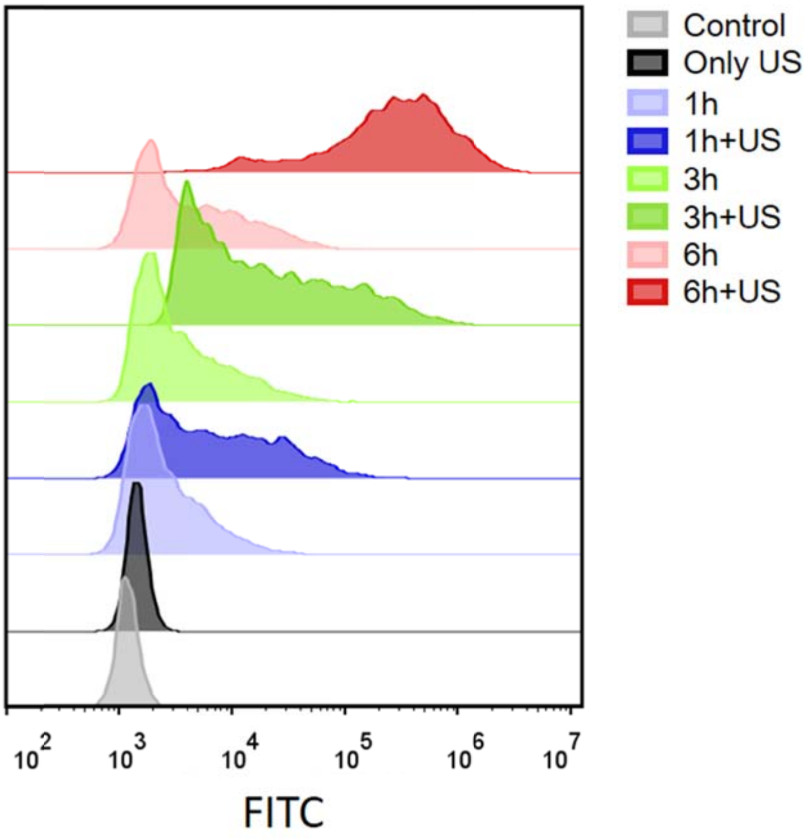


**Figure S19.** ROS generation of WAGQD-$C_{96}$ with different incubation time probed by DCFH-DA with/without ultrasound.

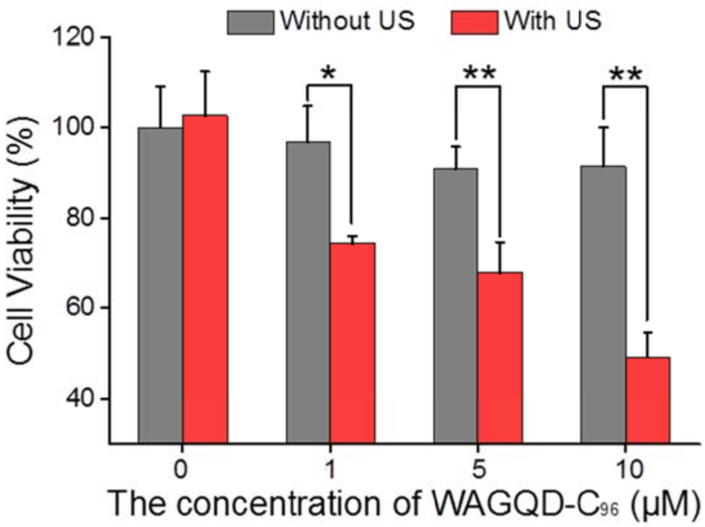


**Figure S20.** Cell viability of HepG2 cells incubated with different concentrations of WAGQD-$C_{96}$ for 24 h with/without ultrasound treatment at 0.56 W/cm$^2$ for 5 min. All data present as means ± SD (n = 4). P-values are calculated using Two-tailed t-test

with GraphPad Prism Software, *, $p < 0.05$; **, $p < 0.01$.)

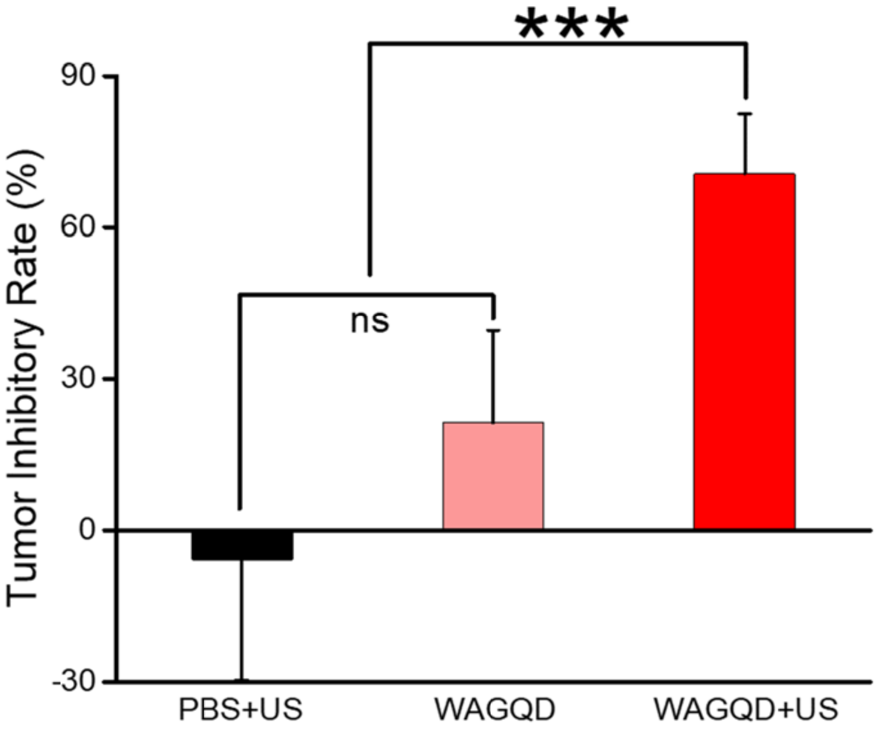


**Figure S21.** The tumor inhibitory rate of PBS+US, WAGQD-$C_{96}$ and WAGQD-$C_{96}$+US groups. All data present as means ± SD (n = 8). P-values are calculated using Two-tailed t-test with GraphPad Prism Software, ns: $p > 0.05$; ***, $p < 0.001$.

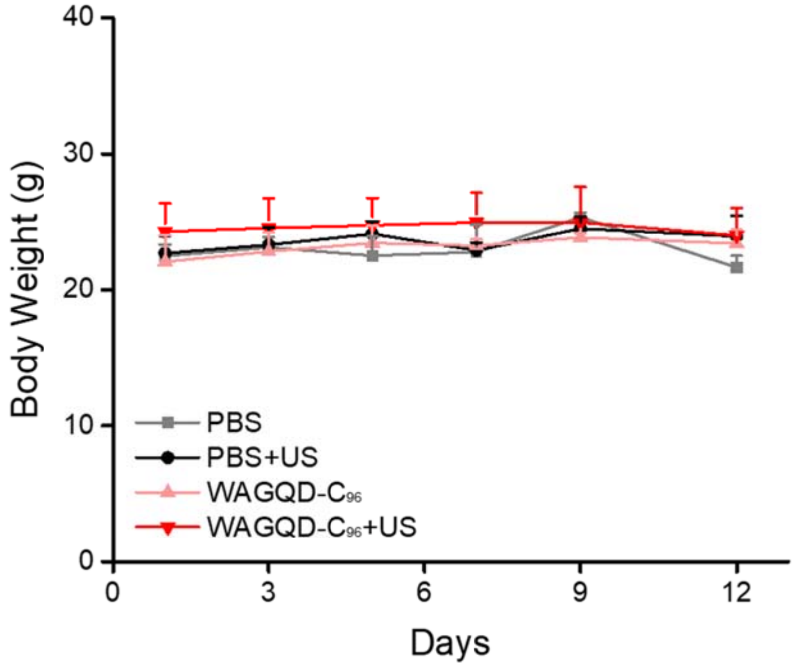


**Figure S22.** Bodyweight of all groups at different time points. All data present as means ± SD (n = 8).

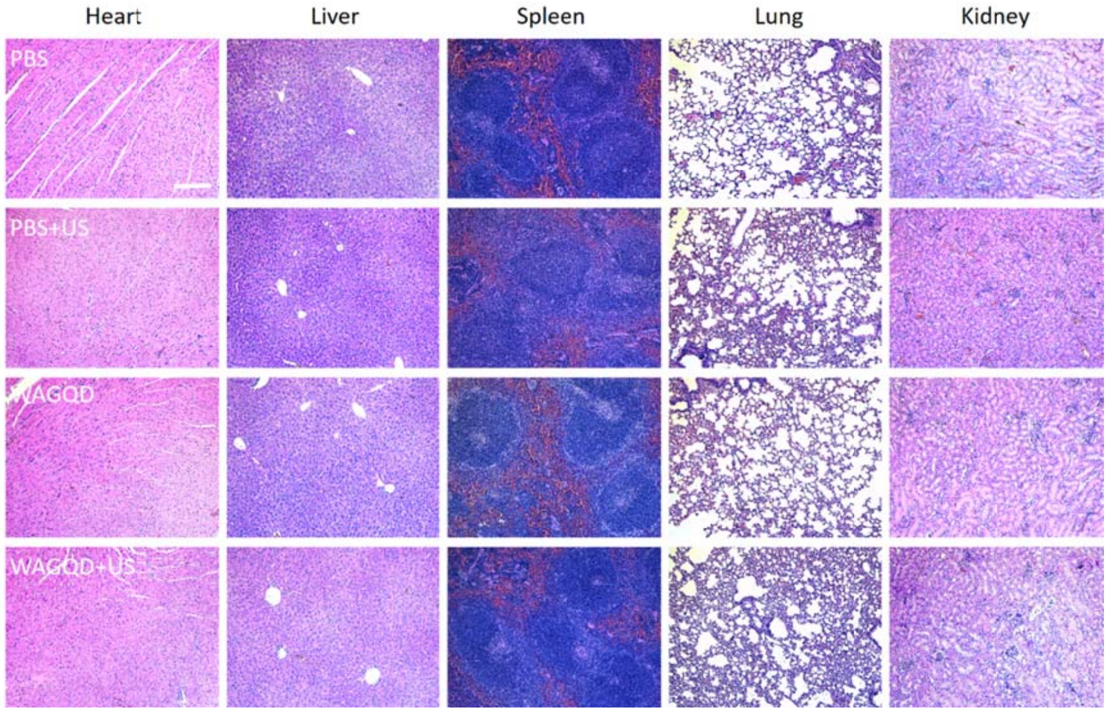


**Figure S23.** H&E staining of major organs for PBS, PBS+US, WAGQD-$C_{96}$ and WAGQD-$C_{96}$+US groups.

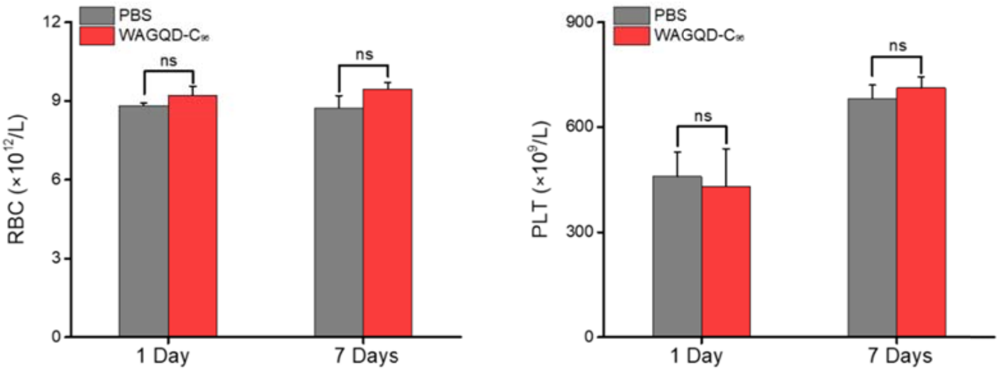


**Figure S24.** The whole blood cell analysis of PBS, PBS+US, WAGQD-$C_{96}$ and WAGQD-$C_{96}$+US groups. All data present as means ± SD (n = 3). P-values are calculated using Two-tailed t-test with GraphPad Prism Software, ns: $p$ >0.05.

## 4. Supporting reference

[1] V. S. Iyer, M. Wehmeier, J. D. Brand, M. A. Keegstra, K. Müllen, *Angew. Chem. Int. Ed.* **1997**, *36*, 1604.

[2] X. J. Zhao, H. Hou, X. T. Fan, Y. Wang, Y. M. Liu, C. Tang, S. H. Liu, P. P. Ding, J. Cheng, D. H. Lin, C. Wang, Y. Yang, Y. Z. Tan, *Nat. Commun.* **2019**, *10*, 3057.

[3] K. Ikemoto, R. Kobayashi, S. Sato, H. Isobe, *Angew. Chem. Int. Ed.* **2017**, *56*, 6511.
